# Investigating executive functions in children with severe speech and movement disorders using structured tasks

**DOI:** 10.3389/fpsyg.2014.00992

**Published:** 2014-09-08

**Authors:** Kristine Stadskleiv, Stephen von Tetzchner, Beata Batorowicz, Hans van Balkom, Annika Dahlgren-Sandberg, Gregor Renner

**Affiliations:** ^1^Department of Psychology, University of OsloOslo, Norway; ^2^Section of Paediatric Neuro-habilitation, Department of Clinical Neurosciences for Children, Oslo University HospitalOslo, Norway; ^3^CanChild Centre for Childhood Disability Research, McMaster UniversityHamilton, Canada; ^4^Behavioural Science Institute, Radboud University NijmegenNijmegen, Netherlands; ^5^Department of Psychology, Gothenburg UniversityGothenburg, Sweden; ^6^Catholic University of Applied SciencesFreiburg, Germany

**Keywords:** executive functions, assessment, aided communication, cerebral palsy, severe speech and movement disorder

## Abstract

Executive functions are the basis for goal-directed activity and include planning, monitoring, and inhibition, and language seems to play a role in the development of these functions. There is a tradition of studying executive function in both typical and atypical populations, and the present study investigates executive functions in children with severe speech and motor impairments who are communicating using communication aids with graphic symbols, letters, and/or words. There are few neuropsychological studies of children in this group and little is known about their cognitive functioning, including executive functions. It was hypothesized that aided communication would tax executive functions more than speech. Twenty-nine children using communication aids and 27 naturally speaking children participated. Structured tasks resembling everyday activities, where the action goals had to be reached through communication with a partner, were used to get information about executive functions. The children (a) directed the partner to perform actions like building a Lego tower from a model the partner could not see and (b) gave information about an object without naming it to a person who had to guess what object it was. The executive functions of planning, monitoring, and impulse control were coded from the children's on-task behavior. Both groups solved most of the tasks correctly, indicating that aided communicators are able to use language to direct another person to do a complex set of actions. Planning and lack of impulsivity was positively related to task success in both groups. The aided group completed significantly fewer tasks, spent longer time and showed more variation in performance than the comparison group. The aided communicators scored lower on planning and showed more impulsivity than the comparison group, while both groups showed an equal degree of monitoring of the work progress. The results are consistent with the hypothesis that aided language tax executive functions more than speech. The results may also indicate that aided communicators have less experience with these kinds of play activities. The findings broaden the perspective on executive functions and have implications for interventions for motor-impaired children developing aided communication.

## Introduction

Executive functions are understood, not as a unitary function but as a psychological construct defined as a set of interrelated high-level cognitive skills that are necessary for purposeful, goal-directed activity (Stuss, 1992; Anderson, 2008; Wiebe et al., 2008; Willoughby and Blair, 2011; Miyake and Friedman, 2012; Benson et al., 2013; Usai et al., 2013). There is a consensus that executive functioning is central in cognitive skills like planning, monitoring results, updating, shifting, and inhibition (Kinsella et al., 2007; Böttcher et al., 2010; Miyake and Friedman, 2012). Planning involves the ability to establish a sequence of sub goals in order to achieve a larger predetermined goal (Hudson and Farran, 2011). Monitoring, or updating (Miyake and Friedman, 2012), involves constant supervision of tasks, with rapid addition and fading of content in working memory. Working memory is the part of the memory system that temporary holds information during mental operations (Eysenck and Keane, 1990; Hitch and Towse, 1995). Inhibition involves overriding of “automatic” behaviors when they are not appropriate (Doebel and Zelazo, 2013). The age at which executive functions emerge is still under debate, but important developments seem to take place from the age of 3 to 4 years (Brocki and Bohlin, 2004; Doebel and Zelazo, 2013). This has been partly attributed to the emergence of language, which broadens the child's ability to reflect on and reason about the world (Astington and Hughes, 2013). Executive functions are related to daily life skills, academic success, and social functioning (Ganesalingam et al., 2011; Foy and Mann, 2013), and it is therefore important to gain knowledge about how these functions develop in typical and atypical populations. Investigations of atypical development may broaden the understanding of the complex relationships between nature and nurture that drives development (Sameroff, 2010).

According to Luria (1961) and Vygotsky (1986), children's private speech in early childhood helps them in solving difficult tasks. Speech takes on a directing and planning function, and contributes to regulating behavior. Private speech was viewed as a forerunner for inner speech, which is an instrument of the thought process. Later research has confirmed that language plays a role in the development of executive functions, and that the ability to verbalize and name objects supports the performance of executive tasks (Miyake et al., 2004; Landry et al., 2012; Doebel and Zelazo, 2013). However, some children do not develop speech due to severe motor impairments and have to use other means of communication. “Aided communication” is defined as the use of communication aids with graphic symbols (like Pictograms, Picture Communication Symbols, and Blissymbols) or letters and words for face-to-face communication. Graphic symbols and words are used in communication aids, e.g., boards, books, and electronic devices with artificial speech output (von Tetzchner and Martinsen, 2000). The aid vocabulary is organized thematically and hierarchically and the user may have to navigate through several pages to indicate the intended expression(s). Children and adults using aided communication are referred to as “aided communicators” (von Tetzchner and Basil, 2011).

Aided communication is chosen when a child's motor impairment is so severe that the use of speech and manual signs is precluded. Severe motor problems may imply very limited voluntary control over physical actions, including movement of the eyes, the head, the arms, and the legs. Depending on their physical abilities, aided communicators access communication aids either directly or with scanning (Light and Drager, 2007). Direct selection involves any form of pointing, for instance with hand, finger, or eye gaze. Specialized computers with eye gaze technology can detect where on the screen the child is looking (see Higginbotham et al., 2007). Selection with scanning may be independent with the use of switches to control item selection, or with the assistance of a communication partner (partner assisted scanning). If possible, direct selection is the preferred mode of operating a communication aid as this is faster than scanning (Ratcliff, 1994; Light and Drager, 2007). Still, regardless of the access method, it may take aided communicators 1 min or more to name a known object with a single symbol, compared to about 1 s in naturally speaking children (von Tetzchner et al., 2012).

The processes related to constructing utterances with natural speech and graphic symbols are very different. Naturally speaking children produce words with relatively little attention to the articulation itself. The articulation process is automatized and usually requires little monitoring, but problems with speech fluency—when the process from conceptualization to spoken articulation is not running smoothly—has been found to be related to executive problems (Engelhardt et al., 2013). For aided communicators, constructing or “articulating” an utterance involves navigating, using direct selection or scanning, on a communication board or an electronic device with several pages to find and indicate one or more graphic symbols (von Tetzchner and Martinsen, 2000). The aided communicator has to remember the location and find and indicate the graphic symbol(s) expressing the intended meaning. Efficient navigation presupposes knowledge of the structure and organization of the graphic symbols in the communication aid. When constructing aided utterances as fast and precisely as possible, the ability to plan the utterance, monitor the progress, and avoid unnecessary detours through the aid's hierarchical system is important (Oxley, 2003; Murray and Goldbart, 2011; Thistle and Wilkinson, 2013).

The role of speech in regulating behavior has mostly been studied in relation to how children regulate their own actions by using their own speech, and when spoken to Fatzer and Roebers (2012); Landry et al. (2012); Doebel and Zelazo (2013). How children using aided communication first express and then internalize private language expressions is not known, neither how they regulate their own behavior and the behavior of others through language, as performing complex actions to reach a goal might be unavailable for them due to their physical impairments. However, language can also be used to regulate the behavior of other people and a child may use language to make others perform actions to reach a particular goal. In such situations, the child's effective use of language implies the use of executive functions. Exploring how young naturally speaking and aided communicators make plans, monitor progress and avoid impulsive errors while using language to direct the actions of others to obtain a goal may therefore give insight into the relationship between language and executive functions.

Using a communication aid requires conscious navigation and deliberate monitoring and the motor impairments of many aided communicators tend to prevent automation of the selection process, thereby placing a constant demand on working memory (Oxley, 2003) and other aspects of executive functioning. Executive functions are generally involved in the construction of aided utterances but the demand on them may vary with communication mode. When an aided communicator is using graphic symbols in a communication book to construct an utterance, the demands on working memory, planning and monitoring will be high, and the avoidance of impulsive errors may be difficult. Also utterances constructed with letters may take a long time to produce but the need for organization and planning might be less with spelling than with graphic symbols because the number of letters is limited and the letters are usually visible all the time.

However, it is usually not only expressive language construction that is affected in aided communicators. Severe physical impairments may make aided communicators unable to reach a physical goal with their own motor acts. Their only means of acting on the physical world may be through instructing other people to perform the actions, that is, by using language for action (Batorowicz et al., 2013). Language may thus have a more decisive role in play and other activities for children with severe motor impairments than for children without such difficulties.

Motor impairment may influence a child's experiences in several ways. Studies show that hands-on experience also contributes to children's regulation of their behavior. One study found that learning to say name shapes like “rhomboid” and “triangle” was not sufficient to make children differentiate between them; they needed the additional information that was gained from touching the shapes (Luria, 1961). Children's participation in social interaction is also believed to influence the development of higher mental functions (Vygotsky, 1986). Children with severe motor and speech impairments have both less experience with handling physical objects than their peers and fewer social experiences (Caillies et al., 2012). In conversations involving aided communicators, the communication partner often takes the initiative, decides the topic and asks questions that only require yes and no answers (von Tetzchner and Grove, 2003; Falkman et al., 2005; Ferm et al., 2005; Clarke et al., 2012). The developmental consequences of these experiences are not known, but from a neuroconstructivist perspective (Mareschal, 2011; Böttcher, 2012) it seems likely that the developmental trajectories of cognition, language, and social functions in the children are negatively affected.

For aided communicators, executive functioning thus seems important both in the construction of utterances and when striving to reach action goals through the use of instructional language. However, there is very little research on executive functioning in this group, and the consequences severe speech and movement disorders may have for the development of executive functions is not known. Indeed, cognitive functioning in general has been little studied in this group, apart from studies looking at the prevalence of intellectual disability (e.g., Andersen et al., 2008; Beckung et al., 2008; Sigurdardottir et al., 2008). Studies investigating different cognitive functions in children with severe speech and movement disorders are therefore needed.

Studies of less motor disabled and mainly speaking children with cerebral palsy (CP) have found that tasks that make demands on executive functioning may be challenging (White and Christ, 2005; Jenks et al., 2007; Böttcher et al., 2010; Pirila et al., 2011; Whittingham et al., 2014) but with some variation with regard to which functions that are affected. Working memory has been found to be reduced, but not inhibitory control (Caillies et al., 2012). However, these studies have rarely included the third of the CP population who are severely motor impaired and in need of aided communication (Andersen et al., 2010). The few studies of severely speech and motor impaired children have focused on attention and working memory, and have found that their attention was reduced compared to peers matched for mental age (Dahlgren et al., 2010), that visual and spatial working memory but not phonological memory was affected (Larsson and Dahlgren Sandberg, 2008), and that working memory capacity increased less than expected from 6 to 12 years (Dahlgren Sandberg, 2006). Several authors have discussed the role of working memory and executive functioning in aided communicators (Light and Lindsay, 1991; Ratcliff, 1994; Oxley and Norris, 2000; Oxley, 2003; Murray and Goldbart, 2011) but to our knowledge there are still no empirical studies of other executive functions than working memory. Indeed, the effectiveness of the aided communication process itself has hardly been investigated (Novak et al., 2013) and little is known about how aided communicators construct utterances that are more complex than when making binary choices such as choosing between milk and juice or between listening to music and watching a video (Murray and Goldbart, 2009). There are studies of adults with aphasia showing that executive functions influence strategy use in communication tasks (Purdy and Koch, 2006), including the learning of graphic symbols (Nicholas et al., 2011), but studies of adults with aphasia may have limited relevance for understanding the development of executive functioning in children with motor impairment.

Measuring executive functions is usually done by either neuropsychological assessment (Lezak, 2004; Strauss et al., 2006), questionnaires (Egeland and Fallmyr, 2010; McCoy et al., 2011) or behavioral tasks (Bechara et al., 1994; Carlson, 2005), or a combination of these. Assessing executive functions in children with severe speech and movement disorders with neuropsychological instruments meets with some challenges, as tests that require the ability to draw, manipulate objects or give a rapid verbal or motor responses cannot be used when the children are unable to perform such actions (Schiørbeck and Stadskleiv, 2008). The tests would therefore need to be adapted, for instance by altering response modality (Alant and Casey, 2005). No validated versions of adapted neuropsychological measures of executive functions exist as of today. Questionnaires such as Behavior Rating Inventory of Executive Functions (Gioia et al., 2000) presupposes that the child can move and talk without restraint. It is therefore necessary to find ways of assessing executive functions that are suitable for children with severe speech and movement impairments (Clarke et al., 2012).

There is a long tradition of using behavioral tasks for exploring aspects of executive functions (see Carlson, 2005), and it has been argued that such tasks may reflect real-life functioning better than test items (Bechara et al., 1994). When using tasks to investigate executive functions in aided communicators, it is important that the tasks draw on the child's best skill, which is language.

The present study thus investigates executive functioning in children with severe speech and movement disorders by using two tasks that resemble everyday activities that require executive functions. In the first task, the child instructs a partner to perform a complex set of actions, such as building a tower of blocks, instead of the child himself executing the actions. In the second, the child is instructed to describe, but not name, objects. Together, these tasks require planning, monitoring of the progress of the task, and avoiding impulsive errors. The executive functions that are required to plan how to instruct the partner so that the building of the tower goes as efficiently as possible, the monitoring of the construction process that is required to correct any misunderstandings and the need to inhibit the impulse to name the objects, thereby breaking the rules of the task, are comparable to the executive functions that are tapped using tests like tower tests and the Stroop test (see Delis et al., 2001). With this approach, executive functions are investigated through language instead of the performance of physical actions. This also reduces the problems encountered when trying to adapt standard neuropsychological tests to children with severe speech and movement impairment.

The performance of the aided communicators is compared to that of typically developing and naturally speaking peers. It was hypothesized that the aided communicators would have more problems and errors than the comparison group, as a consequence of the aided group having a double load on executive functions when performing everyday tasks through others, that is, executive functions both in planning, organizing, and monitoring the tasks itself, and simultaneously in constructing aided utterances. It was further hypothesized that the aided communicators using graphic symbols would have more difficulties than the children using spelling as their communication mode, as the demands on planning and organization is assumed to be higher when navigating through a communication aid than when spelling.

## Materials and methods

This study is part of the international project “Becoming an aided communicator (BAC). Aided language skills in children aged 5–15 years—a multi-site and cross-cultural investigation,” which involves children from 16 countries (von Tetzchner et al., 2012). The present study reports on the performance of aided and naturally speaking communicators in Norway, Canada, the Netherlands, Sweden, and Germany on tasks that they solve in dialog with a communication partner.

### Participants

The children using aided communication were recruited with the help of professionals in the specialized healthcare system and special education system in each of the regions. A search was made for all the high-functioning aided communicators who met the following criteria: (a) were between ages 5 and 15; (b) had used communication aids for a minimum of 1 year; (c) had normal hearing and vision (with corrective technology); (d) were not considered intellectually impaired by their teacher; (e) did not have a diagnosis in the autism spectrum; (f) had speech comprehension considered adequate or near adequate for their age; and (g) speech production was absent or very difficult to understand.

The comparison children were recruited from the class of the aided communicator or from the closest school in the same type of neighborhood (e.g., rural or urban) if the aided communicator went to a special school. The comparison child had the same gender and was the student closest in date of birth to the aided communicator. All children in the comparison group were using speech as communication mode, were healthy, had normal vision and hearing, had no known learning disabilities and attended mainstream schools.

Twenty-nine children using aided communication and 27 typically developing children participated. 20 of the children were Norwegians, 15 Canadian, 12 from the Netherlands, five Swedish, and four from Germany. The age of the children spanned from 6;7 to 15;11 years. Eleven children in each of the groups were boys (see Table 1). There was no significant difference between the aided and the comparison group on age or gender. Twenty-seven of the aided communicators had a diagnosis of CP. For the remaining two children the diagnosis was unknown.

**Table 1:** **Characteristics of the participants**.

	**Aided**	**Comparison**	***t***	***p***
	**Mean (*SD*)**	**Mean (*SD*)**		
Age, months (*N* = 29, 27)	136.1 (33.7)	137.1 (31.5)	−0.114	0.910
Gender, boys % (*N* = 29, 27)	37.9 (49.4)	40.7 (50.1)	−0.211	0.834
TROG-2, z-scores (*N* = 28, 14)	−1.50 (1.22)	−0.29 (0.99)	−3.434	0.002^*^
BAC Naming, correct out of 20 items (*N* = 23, 14)	14.2 (5.5)	19.6 (0.6)	−4.641	0.000^*^
BAC Naming, mean time in sec (*N* = 20, 14)	42.8 (32.0)	1.8 (0.7)	5.568	0.000^*^

t, t-test (independent samples t-test, equal variances not assumed).

* p < 0.05.

Language comprehension was assessed with the Test for the Reception of Grammar, second edition (TROG-2) using national norms (Bishop, 2003) (see Table 1). The test places small demands on motor skills; the child indicates which of four pictures that corresponds to the sentence spoken. If the child is unable to point with the hand, a partner used assisted scanning. This implies that the four pictures were pointed at in a systematic manner and the child indicated “yes” or “no” for each picture (Schiørbeck and Stadskleiv, 2008). The mean scores were below the age mean, but within two standard deviations of the mean, for both groups. The difference between the groups was significant.

Naming and expressive language speed was compared using *BAC Naming*. The task of the child is to name drawings of 20 objects and animals, which are shown one at a time. The aided communicators correctly named on average 14.2 (71%) of the drawings and the comparison group 19.6 (98%), a significant difference. The aided group used significantly longer time giving correct names than the comparison group. There is a significant and positive correlation (*r* = 0.60) between scores on TROG-2 and number of correct answers on BAC Naming.

A description of the aided communicators is given in Table 2. Gross and fine motor functions were classified according to the Gross Motor Classification System (GMFCS) (Palisano et al., 1997) and Manual Ability Classification System (MACS) (Eliasson et al., 2006). They both have five levels with level I indicating best possible functioning and level V severe difficulties. On level I of GMFCS, the child walks without assistance, while on level V the child cannot sit or stand independently. On level I of MACS, the child handles objects easily and successfully, while on level V the child is unable to handle objects. The median scores of the aided communicators were level V on both GMFCS and MACS.

**Table 2:** **Characteristics of the aided communicators**.

**Variable**	**Specification**	**Aided group**		***t***	***p***
		**Symbols users**	**Spellers**		
Gender, boys % (*N* = 15, 14)	Mean (*SD*)	26.7 (45.8)	50.0 (51.9)	−1.281	0.212
Age, in months (*N* = 15, 14)	Mean (*SD*)	120.5 (37.0)	152.9 (19.6)	−2.972	0.007^*^
GMFCS level (*N* = 15, 14)	Level I (n)	1	0		
	Level II (n)	0	0		
	Level III (n)	2	0		
	Level IV (n)	5	4		
	Level V (n)	7	10		
MACS level (*N* = 15, 14)	Level I (n)	1	0		
	Level II (n)	1	0		
	Level III (n)	2	0		
	Level IV (n)	4	4		
	Level V (n)	7	10		
Viking speech scale level (*N* = 15, 14)	Level I (n)	0	0		
	Level II (n)	0	0		
	Level III (n)	1	0		
	Level IV (n)	14	14		
CFCS level (*N* = 11, 12)	Level I (n)	0	0		
	Level II (n)	3	10		
	Level III (n)	6	2		
	Level IV (n)	2	0		
	Level V (n)	0	0		
Communication access (*N* = 10, 10)	Direct (hand) (n)	7	1		
	Direct (gaze) (n)	2	2		
	Scanning, switches (n)	1	7		
TROG-2, z-scores (*N* = 15, 13)	Mean (SD)	−2.09 (1.04)	−0.82 (1.08)	−3.154	0.004^*^
BAC Naming, correct of 20 items (*N* = 11, 12)	Mean (SD)	9.7 (4.3)	18.3 (2.4)	−5.749	0.000^*^
BAC Naming, time in sec (*N* = 10,10)	Mean (SD)	49.3 (43.6)	36.4 (17.5)	0.873	0.400

t, t-test (independent samples t-test, equal variances not assumed).

* p < 0.05.

The Viking Speech Scale (Pennington et al., 2010) has four levels, with level I indicating normal function and level IV being used for children with no functional speech. The median scores of the aided communicators was level IV. The Communication Functioning Classification system (CFCS) (Hidecker et al., 2011) has five levels with level I indicating best functioning and level V the most affected. On level I the child can communicate efficiently and without reduced speed with both known and unknown partners, on level II the child can communicate efficiently with both familiar and unfamiliar partners, but the speed of communication is reduced compared to peers, on level III the child can communicate effectively with known partners, but not with unknown, on level IV the child can communicate somewhat efficiently with known partners, while children on level V have difficulties with being understood even by familiar partners. The aided communicators had scores between II and IV, with a median score of II for the spellers and III for the symbol users.

Fourteen of the 29 children used spelling, either alone or in combination with graphic symbols, while 15 used only graphic symbols. Most of the symbol users accessed their communication devices directly by pointing with hand or eye gaze. Of the spellers, scanning with two switches (i.e., step scanning, with one switch used to progress between items and a second to select the item) was most common. There were no significant differences between children using symbols and children using spelling concerning gender and speed of naming objects, but the spellers were on average more than 2 years older, showed better comprehension of language and named significantly more objects correctly than the non-spellers.

Parents, peers, and teachers of the aided and the comparison group were asked to participate as communication partners in the study. The children in the aided group and the comparison group were asked to nominate a peer with whom they wanted to do the tasks. The peers were friends whom they knew well, and the aided communicators and the peers had experience in communicating together. Some of the children in the aided group were unable to nominate a friend, and a sibling was asked instead. The parents of the all the participating children (aided group, comparison group and peers) gave consent to their child's participation.

### Tasks

The two tasks *BAC Construction* and *BAC Description without naming* were used to obtain measures of planning, monitoring, and impulsivity. The performance on the tasks was videotaped and the dialog was transcribed in accordance with existing standards for such transcriptions (see von Tetzchner and Basil, 2011). A coding system was developed based on a detailed analysis of the videos of four children, each interacting with three different partners. Inter-rater reliability was established by two independent raters who watched the videos and scored all tasks until full agreement was reached.

#### BAC Construction

In BAC Construction the child has a physical model in a box, placed so that the model is not visible to the partner. The child instructs the partner to construct the same figure. There are two training items (loading a lorry) and eight task items (dressing a doll, making a necklace of beads, building a tower of Lego blocks, and laying a pattern of domino pieces). The partner has many more clothes, beads, Lego blocks, and domino pieces than are needed for construction. To reduce the memory load of the child, the model was visible to the child throughout the task.

Table 3 shows the measures used in the analysis: correctly solved items, the time it took to solve the items, the child's planning ability, as well as the child's monitoring of the construction process (see also Batorowicz et al., 2013, 2014).

**Table 3:** **Task measures**.

**Task**	**Measures**	**Categories**		**Scores**
BAC Construction	Items correctly solved	Items constructed correctly		0–100%
	Time	Average time use per item (sec)		
	Planning	Difficult to decide if the child has a plan		0
		No evidence of a plan		1
		Plan evolved during solution of task		2
		Clear plan from the beginning		3
	Object	Proportion of necessary objects named	Less than necessary number of objects mentioned	<1.00
			Necessary number of objects mentioned	1.00
			More than necessary number of objects mentioned	>1.00
	Attributes	Proportion of necessary attributes named	Less than necessary number of attributes mentioned	<1.00
			Necessary number of attributes mentioned	1.00
			More than necessary number of attributes mentioned	>1.00
	Specificity	Wrong description		1
		Too low specificity (only correct object or attributes)		2
		Perfect specificity (correct object and attributes)		3
		Too high specificity (correct object, one attribute too many)		4
		Much too high specificity (correct object, too many attributes)		5
	Monitoring	Specificity provided at the start of the item subtracted from specificity provided after watching the partner constructing		0.0–5.0
BAC Description	Items correctly solved	Items named correctly by partner		0–100%
	Impulsivity	Items the child names, contrary to instruction		0–100%

*Items solved correctly* are items where the partner constructed an exact copy of the child's figure. Almost similar models, like Lego towers with one block in the wrong color, were scored as failed.

*Planning* is defined as the type of strategy that could be observed when the child provided instructions to the partner. The quality of the child's planning was classified on a scale from 0 to 3. A score of 0 means that it was difficult to decide if the child had a plan, a score of 1 means that the child did not seem to have a clear plan (e.g., if the child started the Lego item by describing a block positioned in the middle of the tower), a score of 2 means that the child initially did not seem to have a clear plan, but that a plan seemed to emerge during the item performance (e.g., started by having the partner put on shoes before trousers on the doll, but then progressed without problems from there on), and a score of 3 means that the child seemed to have a clear plan throughout the item. A score for the observed planning was given for each of the eight items administered, and the child's average planning score for the whole task was based on this.

A five-point *specificity scale* was used to measure *monitoring*, based on the preciseness of the child's utterance. The measure makes it possible to look not only on the quantity of information provided, but also on the quality of it. A precise description should include both a description of the *object* (such as the type of clothes needed to dress the doll) and the *attributes* of the object (e.g., color of the pants). The number of objects and attributes the child mentioned was compared to the number that was necessary for a precise description. A specificity score of 1 would indicate very low specificity (wrong description), a score of 2 a little too low specificity (only correct object or only correct attributes), a score of 3 a perfect specificity (the objects and attributes needed), a score of 4 a little too high specificity (one attribute too many), and a score of 5 a much too high level of specificity (more than one attribute too many). *Initial specificity* is the score of the content provided by the child before the partner has started constructing, while the *final specificity* is the total content provided by the child while watching the partner constructing the item. If the child did not add any information during the task, the final specificity would be equal the initial. If the child saw the need to add information while watching the partner constructing, the final specificity would be higher than the initial. *Monitoring* is the difference between initial and final specificity. It is a continuous variable ranging from 0 (no new information added) to a maximum of 5 (the maximum specificity score); If the child adds any task-relevant information while observing the partner constructing the item, the monitoring score will be a positive number larger than 0 and smaller than 5.

#### BAC Description without naming

In “BAC Description without naming” (henceforth abbreviated as BAC Description), the child was presented with 12 different drawings of an object and instructed to describe but not name, the objects in such a manner that the communication partner could name the objects. Three of these 12 drawings were training tasks, so that a total of nine drawings are included in the analysis. The drawings were in a box and not visible to the partner who was seated opposite the child. The partner was allowed to make as many guesses as were necessary to name each item, but not to ask any leading questions (like “What is it used for?”). The child could continue to describe the object also after the partner had started to guess. To reduce the memory load of the child, the drawing was visible to the child throughout the task. The three training items and nine task items consisted of common objects like a chair, a book, and an apple. From this task measures of correctly solved items and impulsivity were obtained (see Table 3).

*Correctly solved items* are items where the partner names the object. Items that were almost solved, for example saying “orange” when there was a picture of an apple, were scored as failed, as were items where the partner answered wrong or was not able to make a guess.

*Impulsivity* is when the child names the object. This is a violation of the task instruction as the child was instructed not name the object.

### Ethics

The ethical approval for the study was obtained by each participating site following the national procedures for ethical approval.

### Statistics

Data was coded in IBM SPSS Statistics, version 20. Independent sample *t*-tests were used for comparisons between the aided group and comparison group, and between aided communicators using symbols and letters. Spearman's Rho rank order correlations were used to investigate the relationship between variables.

## Results

There was a significant correlation between age and percentage of correctly solved items on the BAC Construction task, but not on the BAC Description task (see Table 4) when looking at both the aided group and the comparison group together. Verbal comprehension, as measured by results on TROG-2 was not significantly related to percentage of solved items on the BAC Description task, but was related success on the BAC Construction task. Expressive verbal abilities, as measured by number of correct items on the BAC Naming task, were significantly and positively correlated with percentage of solved items both on the BAC Construction task and on the BAC Description task.

**Table 4:** **Correlation between items correctly solved and age, verbal comprehension and expressive verbal abilities**.

	**BAC Construction items correctly solved**	**BAC Description items correctly solved**
Age	0.37^**^	0.27
Verbal comprehension	0.45^**^	0.24
Expressive verbal abilities	0.72^**^	0.46^**^

^*^p < 0.05, Spearman's Rho rank order correlations (two-tailed).

** p < 0.05, Spearman's Rho rank order correlations (two-tailed).

There were differences between the aided group and the comparison group with regards to task success. On BAC Construction, the aided group solved 64.7% of the items and the comparison group 92.6%, a significant difference (see Table 5). The aided communicators took almost five times as long as the naturally speaking children to complete the items on this task. On the BAC Description task, the aided group solved 65.1% and the comparison group 96.7%, a significant difference.

**Table 5:** **Task performance of aided group and comparison group**.

**Tasks and variables**	**Aided**	**Comparison**	***t***	***p***
	**Mean (*SD*)**	**Mean (*SD*)**		
**BAC CONSTRUCTION**				
Items correctly solved (%) (*N* = 28, 27)	64.7 (34.1)	92.6 (10.1)	−4.147	0.000^*^
Time (sec) (*N* = 26,27)	419.6 (251.7)	88.2 (51.7)	6.808	0.000^*^
Planning (*N* = 27, 27)	2.5 (0.5)	2.8 (0.2)	−3.353	0.002^*^
Objects (*N* = 27, 27)	0.58 (0.29)	0.90 (0.10)	−5.384	0.000^*^
Attributes (*N* = 27, 27)	1.01 (0.51)	1.67 (0.55)	−4.665	0.000^*^
Specificity of utterance (initial) (*N* = 27, 27)	2.2 (0.6)	3.2 (0.5)	−6.575	0.000^*^
Specificity of utterance (final) (*N* = 27, 27)	2.6 (0.7)	3.5 (0.5)	−6.029	0.000^*^
Monitoring (*N* = 28, 27)	0.4 (0.2)	0.3 (0.3)	0.693	0.491
**BAC DESCRIPTION**				
Items correctly solved (%) (*N* = 27, 22)	65.1 (28.7)	96.7 (7.4)	−5.517	0.000^*^
Impulsivity (%) (*N* = 27, 25)	14.6 (23.9)	1.6 (4.4)	2.793	0.009^*^

t, t-test (independent samples t-test, equal variances not assumed).

* p < 0.05.

### Planning, monitoring, and impulsivity in aided communicators and comparison group

A significant group difference in the planning score (see Table 5) indicates that more children in the aided group than in the comparison either did not have a plan from the start or had to develop a plan during the item. The aided group described significantly less objects and attributes than the comparison group. In both groups there was a considerable variation in number of attributes mentioned, with means ranging from 0.28 to 2.76 in the aided group and from 0.78 to 2.79 in the comparison group. (A proportion larger than 1.00 indicates that more attributes than necessary were described.) In the aided group, the initial and the final specificity were significantly below the initial and final specificity in the comparison group. The increase in specificity from initial to final was equal in both groups, implying that there was no group difference with regards to monitoring. On the BAC Description task, there was significantly more evidence of impulsivity in the aided group than in the comparison group.

### Planning, monitoring, and impulsivity in relation to communication mode

On the BAC Construction and the BAC Description tasks the percentages of solved items were significantly lower for aided communicators using graphic symbols than for those using spelling (see Table 6). There was a significant difference between the two groups in planning and specificity of the utterances, but not on monitoring or degree of impulsivity.

**Table 6:** **Task performance of aided communicators using symbols and spelling**.

**Tasks and variables**	**Symbols Mean (*SD*)**	**Spelling Mean (*SD*)**	***t***	***p***
**BAC CONSTRUCTION**				
Items correctly solved (%) (*N* = 15, 13)	43.9 (33.2)	88.6 (12.9)	−4.810	0.000^*^
Time (sec) (*N* = 15, 13)	353.7 (233.0)	495.6 (259.8)	−1.511	0.144
Planning (*N* = 14, 13)	2.2 (0.5)	2.8 (0.4)	−3.161	0.004^*^
Objects (*N* = 14, 13)	0.44 (0.29)	0.74 (0.20)	−3.131	0.005^*^
Attributes (*N* = 14, 13)	0.92 (0.44)	1.10 (0.57)	−0.950	0.352
Specificity (initial) (*N* = 14, 13)	1.9 (0.6)	2.6 (0.5)	−3.637	0.001^*^
Specificity (final) (*N* = 14, 13)	2.2 (0.7)	2.9 (0.5)	−3.022	0.006^*^
Monitoring (*N* = 14, 13)	0.4 (0.2)	0.3 (0.2)	0.733	0.471
**BAC DESCRIPTION**				
Items correctly solved (%) (*N* = 13, 14)	52.5 (26.1)	76.8 (26.6)	−2.402	0.024^*^
Time (sec) (*N* = 4, 8)	234.2 (177.8)	247.9 (139.6)	−0.134	0.898
Impulsivity (%) (*N* = 13, 14)	20.1 (20.0)	9.6 (26.7)	1.162	0.257

t, t-test (independent samples t-test, equal variances assumed).

* p < 0.05.

### Executive functioning and task success

Aspects of executive functioning correlated significantly with task success when the results for both groups were combined (see Table 7). In the combined group (aided communicators and comparison group) there was a significant positive correlation between planning and number of correct items on the tasks and a negative correlation between impulsivity and percentage of correct items on both tasks. Monitoring and performance on the BAC Description task was significantly and negatively associated in the aided group and in the combined group.

**Table 7:** **Correlation between executive functions and items correctly solved on BAC Construction and BAC Description**.

	**Aided group**		**Comparison group**		**Groups combined**	
	**Items correctly solved**		**Items correctly solved**		**Items correctly solved**	
	**BAC construction**	**BAC description**	**BAC construction**	**BAC description**	**BAC construction**	**BAC description**
Planning	0.64^**^	0.37	0.26	0.19	0.54^**^	0.42^**^
Monitoring	0.16	−0.46^*^	0.08	−0.34	0.05	−0.38^**^
Impulsivity	−0.34	−0.63	−0.42	−0.88^**^	−0.39^**^	−0.69^**^

* p < 0.05, Spearman's Rho rank order correlations (two-tailed).

** p < 0.01, Spearman's Rho rank order correlations (two-tailed).

### Executive functioning and verbal abilities

Planning was positively related to verbal comprehension and expressive verbal abilities in the aided group, but not in the comparison group (see Table 8). Monitoring and impulsivity were not related to verbal comprehension or expressive verbal abilities, neither in the aided group nor the comparison group alone, or in the groups combined.

**Table 8:** **Correlation between executive functions and language comprehension and expressive language skills**.

	**Aided group**		**Comparison group**		**Groups combined**	
	**Language comprehension**	**Expressive verbal abilities**	**Language comprehension**	**Expressive verbal abilities**	**Language comprehension**	**Expressive verbal abilities**
Planning	0.40^*^	0.60^**^	−0.30	0.38	0.38^*^	0.54^**^
Monitoring	0.09	0.03	−0.33	0.07	0.09	0.05
Impulsivity	−0.13	−0.26	0.22	0.21	−0.21	−0.33

* p < 0.05, Spearman's Rho rank order correlations (two-tailed).

** p < 0.01, Spearman's Rho rank order correlations (two-tailed).

## Discussion

Both the aided group and the comparison group completed most of the items in the BAC Description and BAC Construction tasks correctly. In spite of their severe motor disabilities and lack of speech, the aided communicators were able to plan, execute and monitor instructions to make partners perform the physical acts needed to construct something the partners could not see. They were also able to describe objects in such a way that the partners, who could not see the object, were able to name them. This demonstrates how language use can compensate for the lack of motor skills that are required to act directly on the physical world and the achievements of the motor-impaired aided communicators. However, the considerable time and effort the aided communicators needed to complete the tasks compared to their typically developing peers imply a continuous demand on executive functions.

The aided group was hypothesized to have more difficulties with the tasks because planning both the language construction and the complex set of actions for the partner to perform represents a double demand on executive functions. The naturally speaking children only had to plan the actions as articulation of speech was automatized and required little cognitive effort. It was also hypothesized that the aided communicators using spelling would perform better than the aided communicators using graphic symbols, because finding and selecting symbols in a communication book or electronic communication aid place larger demands on executive functions than spelling. Both of these hypotheses were supported: the aided communicators solved significantly fewer tasks than the comparison group and the graphic symbol users solved significantly fewer tasks than the spellers. The results indicate that although language use can compensate for being unable to perform a complex set of actions to reach a physical goal (language for action), the process of aided language construction, and especially when involving the use of graphic symbols, was taxing the children's overall executive capacity.

The ability to make a clear plan was positively related to outcome on both the tasks for the groups combined, and on the BAC Construction task in the aided group alone. This may reflect that for aided communicators the creation of an initial plan and how they communicate this from the start plays a greater role for task success than in naturally speaking children who can correct mistakes more easily while monitoring the construction process. The results, specifically the scores on number of objects and attributes mentioned and the specificity scores show that on average the comparison group provided more information than the aided group. This may reflect the ease of articulation of speech compared to constructing aided utterances. As the specificity scale gives an evaluation of the quality of the information provided, not only of the amount, the results show that the aided communicators provided somewhat imprecise information at the start of the task and then added necessary information, while the comparison group on average provided precise enough information at the start but still added more details while observing the partner's task construction.

Monitoring was defined as the child providing more information to the partner after the initial description and as a result of watching the construction process, and both groups provided the same amount of extra information. However, in the aided group, monitoring was negatively related to outcome on the BAC description task. This negative relationship might reflect that for the children who needed to supply the most extra information on the construction task, the task of describing an object without any support from a communication partner was extra challenging.

Monitoring might also be viewed as an intrinsic part of the aided communication process. However, aided communicators do not have the full responsibility for this because aided language production tend to be co-constructive; that is, the naturally speaking communication partner makes interpretations and guesses during message construction which are confirmed or rejected by the aided communicator (Collins, 1996; von Tetzchner and Grove, 2003). When aided communicators construct a graphic utterance with symbols the communication partner usually interprets the meaning of each symbol, formulates the utterance in speech and seeks acknowledgement of the spoken formulation from the aided communicator. The partners thus function as interpreters and translators. This is true also when the utterance is produced with artificial speech, unless the graphic symbol or symbol sequence produces a pre-made sentence. Moreover, although it is the aided communicator who has to produce the graphic utterance, the communication partners often take a leading role and dominate the co-construction even when the message is about an event that is unknown to them (von Tetzchner and Martinsen, 1996; Collins and Marková, 1999; Batorowicz et al., 2014). Monitoring is thus a core element of the aided language process and this might explain why monitoring was less affected by aided language experience. This also implies that the emergence of language and the language construction process is quite different in aided and naturally speaking communicators and that the many proposed mechanisms in typical language construction and development (see Gerken, 2005) may not apply to the same extent in the construction and development of utterances with communication aids. Utterances with graphic symbols are produced, but may also be processed and represented mentally, in a different manner from speech.

Impulsivity was negatively related to outcome in both groups. There was more evidence of impulsivity in the aided group than in the comparison group, but most of the children in both groups had no problems with inhibiting the impulse to name an object they were instructed not to name. This is in line with findings from other studies, indicating that inhibition is not affected in children with CP (Caillies et al., 2012). There was, however, some variation within the aided group, where the graphic symbol users made somewhat more impulsive errors than the spellers, that is, they named more objects instead of describing them. One reason might be that communication aids with graphic symbols contain a fixed vocabulary that can be used for constructing utterances. A limited number of graphic symbols imply that each symbol may have to be used to construct a broader set of meanings than the corresponding word for speaking children. One result of this may be a co-construction process where aided communicators provide one or two key words and then rely on their communication partner to complete the message (von Tetzchner and Martinsen, 2000; Brekke and von Tetzchner, 2003). Children who are able to spell are less restricted in producing their utterances than graphic symbol users. However, the results also show that with clear instructions and training most of the symbol users were able to provide a richer description than just naming.

Monitoring and impulsivity were not related to language comprehension or expressive language skills, but to overall success on the tasks. Planning was also related to success on the tasks, as well as to language comprehension and expressive language skills. These findings indicate that not only comprehension, but also the quality of the verbal output of the child, correlates with the regulation of behavior. Related findings have been reported in other studies, where expressive language was found to play a role when children were asked to regulate their own behavior (Fatzer and Roebers, 2012; Landry et al., 2012; Doebel and Zelazo, 2013) and verbal fluency in the development of executive functions (Brocki and Bohlin, 2004).

In addition to the demands on planning, the children's ability to use language to regulate another person's behavior may have been influenced by their earlier experiences. Compared to typically developing children, children with motor impairments are likely to have less experience with active involvement in ordinary construction play like dressing a doll or building a tower of blocks or other construction activities (Caillies et al., 2012) and hence have fewer experiences to build on when trying to find ways to do the tasks in an efficient manner. Instructing others may compensate for the child's lack of motor skills but descriptions of child-adult interactions where the child instructs the adult to do something that is unknown to the adult are rare (see von Tetzchner and Martinsen, 1996). Aided communicators are therefore likely to have limited experience with giving others detailed instructions to construct something. The comparison group probably had more experience with construction play. They may also have relatively little experience with this form of instructing actions but explaining to others how to do things is not uncommon among children (for instance in play activities). This finding is in line with the earlier cited finding that verbal skills are not sufficient to solve a task, but that hands-on experience is also needed (Luria, 1961).

The results show that there is more variation in the aided group than in the comparison group. This may indicate that the aided group was more heterogeneous than intended when searching for participants. All the aided communicators were judged by their teachers not to be intellectually impaired and the results on TROG-2 supported this evaluation as the group mean was within two standard deviations from the age mean. It is, however, possible that some of the aided communicators had specific difficulties in some areas which were not discovered by the teachers. There seemed to be some correspondence between communication mode and language level and age. The graphic symbol users were younger than the spellers, which was expected as the expressive language development of aided communicators typically starts with graphic symbols and progress to spelling (although many continue to have problems with reading and writing). They also scored lower on verbal comprehension and naming. However, a thorough assessment of aided communicators is recommended, so that specific cognitive challenges can be detected and taken into consideration in educational planning.

### Suggestions for interventions

Through development there is a complex interplay between neurological impairment and plasticity, cognitive development, and developmentally affording experiences (Böttcher, 2010). Using language for action may compensate for motor impaired children's limited physical and social experiences, but this implies good abilities for planning and organization. One recommendation that emerges from the findings in this study is to support the development of executive functions by giving young aided communicators more opportunities for engaging in construction play and other construction activities. This may support not only executive functions, but also lead to greater autonomy and social participation (Batorowicz et al., 2014).

Furthermore, greater focus may be given to structural stability in aid content to promote increasing automation of language construction in children using aided language. Spelling might reduce the executive demands inherent in the construction process but many nonspeaking children have significant difficulties and delays related to literacy acquisition independent of general cognitive function (Smith, 2005; Larsson and Dahlgren Sandberg, 2008), so this might not be an available option for all aided communicators. It is also notable that the ability to spell did not lead to faster solutions, implying that this form of communication mode is still taxing on the child's cognitive capacity.

### Limitations of the present study

The present study includes a small group of aided communicators and a matched group of typically developing peers. It cannot be ruled out at that the small sample size may have caused a bias in one way or the other, and the findings of the present study will need to be confirmed in future studies.

A search for aided communicators filling the inclusion criteria was made in all the regions included in the study. Although the aim was not to have a complete geographical sample, the experience was that finding the aided comparison children proved harder than anticipated at the start of the study. This might indicate that the group presented in this study is representative for the high functioning aided communicators. For instance, for the Norwegian sample children from the 11 southernmost counties of Norway, where approximately 60% of the country's population live, were included. Previous studies in Norway have indicated that 0.023% of children use graphic communication and that a quarter of these children can be classified as belonging to the expressive group (von Tetzchner, 1997). With a population of 5 100 000, where 12.1% is children between 6 and 15 years of age (Statistics Norway, 2014^1^), it can be estimated that the expressive group in the region included in the study totals approximately 21–22 children. So even though the sample size is small, it probably represents a fair proportion of the eligible children in the geographical area covered.

The measures that were used in this study give insight into the use of executive functions in daily life as the tasks chosen resemble everyday activities that children are likely to encounter, such as building a tower of blocks, making a pattern with beads, matching amounts and telling people about something they have observed but the other person has not. The ecological validity of the tasks is therefore assumed to be high. As we have not employed other methods for investigating executive functions, the study cannot give information about how these measures compare to other measures of executive functions, such as neuropsychological tests.

### Suggestions for further studies

The study has shown that structured tasks resembling everyday activities can give important information about executive functions in a group of children where this information is particularly important and for whom traditional tests and questionnaires cannot be used without adaptations. The tasks required very few skills besides the ability to produce an utterance (in any manner possible and without any time limit) and utilized the area where children with severe motor impairments function best. Further studies should look at the kind of interventions that are provided to this group of children and how these may influence development and learning in aided communicators. In spite of the potential importance of being able to use language to guide another person to perform a specific set of actions, this way of using aided communication is hardly described. An important research issue might be to develop interventions aimed at providing opportunities for creative language construction and active exploration of the environment, and investigate their influence on executive functioning.

The results for the comparison children, who mastered nearly all the items, point to the possibility that the items may have been too easy to give information about executive functions in a group of typically developing 5–15 years olds. For instance, in the Lego task they only needed to describe a model of a tower and not a more challenging three-dimensional model. Future studies should take this into consideration.

Future studies may also include more information about the etiologies of the CP in the children, as previous studies have suggested that there may be differences in cognitive profiles related to subtypes of CP that are not explained by differences in intelligence, but which may be related to the localization of the insult in the brain (Pueyo et al., 2003; van Kampen et al., 2012).

Comparing performance of children with various disabilities and disorders on tasks of the same type as used in this article, including children without motor disabilities and speech impairment, may give information about the role that different forms of language experiences play for planning and other executive functions. It may be useful to substitute some of the easier items within each task with more difficult ones and to compare performance on these items with traditional tests and questionnaires used to measure executive functions.

## Author contributions

Kristine Stadskleiv has been involved in collecting, transcribing, coding, and analyzing the data included in the study and has had the main responsibility for preparing the manuscript. Stephen von Tetzchner is the principal investigator of the study “Becoming an aided communicator” and is responsible for the design of the study, for collecting data from the Norwegian participants, for monitoring the project progress and for preparation of this manuscript. Beata Batorowicz was involved in developing the coding system for the tasks, as well as in analyzing the data and preparing the manuscript. Hans van Balkom, Annika Dahlgren-Sandberg, and Gregor Renner were involved in collecting and analyzing data.

### Conflict of interest statement

The Associate Editors Ayelet Lahat and Oriane Landry declare that, despite being affiliated to the same institution as the author Beata Batorowicz, the review process was handled objectively and no conflict of interest exists. The authors declare that the research was conducted in the absence of any commercial or financial relationships that could be construed as a potential conflict of interest.

## Footnotes

^1^http://www.ssb.no/befolkning/statistikker/folkemengde/aar/2014-02-20#content
